# Genome Sequence of the Photoarsenotrophic Bacterium *Ectothiorhodospira* sp. Strain BSL-9, Isolated from a Hypersaline Alkaline Arsenic-Rich Extreme Environment

**DOI:** 10.1128/genomeA.01139-16

**Published:** 2016-10-13

**Authors:** Jaime Hernandez-Maldonado, Brendon Stoneburner, Alison Boren, Laurence Miller, Michael Rosen, Ronald S. Oremland, Chad W. Saltikov

**Affiliations:** a Department of Microbiology and Environmental Toxicology, University of California Santa Cruz, Santa Cruz, California, USA; b U.S. Geological Survey, Menlo Park, California, USA; c U.S. Geological Survey, Carson City, Nevada, USA

## Abstract

The full genome sequence of *Ectothiorhodospira* sp. strain BSL-9 is reported here. This purple sulfur bacterium encodes an *arxA*-type arsenite oxidase within the *arxB2AB1CD* gene island and is capable of carrying out “photoarsenotrophy” anoxygenic photosynthetic arsenite oxidation. Its genome is composed of 3.5 Mb and has approximately 63% G+C content.

## GENOME ANNOUNCEMENT

Arsenic-rich soda lakes are ideal environments for culturing microorganisms with unique metabolic capabilities for coupling cellular energy production to arsenic oxidation and reduction (1–6). Here, we report the assembled genome of an anoxygenic photosynthetic arsenite-oxidizing (“photoarsenotrophic”) bacterium, *Ectothiorhodospira* sp. strain BSL-9. This microbe was isolated from Big Soda Lake, an arsenic-rich (~25 µM), hypersaline (26 to 88 g/liter total dissolved solids), alkaline (pH 9.7) lake located in Nevada (39°31ʹN 118°52ʹW) (7–9). Moreover, this crater lake has a well-defined seasonal bloom of purple sulfur bacteria (*Chromatium* and *Ectothiorhodospira* species) (10) that are proposed to contribute to the arsenic geochemical cycle.

Assessment of the BSL-9 genome revealed an arsenic gene island, *arxB2AB1CD* (11), which is predicted to encode the arsenite oxidase gene *arxA*. Moreover, *arxB2AB1CD* encodes a [4Fe-4S]-containing protein (*arxB2*), a second [4Fe-4S]-containing protein (*arxB1*)*,* a membrane protein (*arxC*)*,* and a TorD-like protein involved in molybdenum enzyme biogenesis (*arxD*). In the chemoautotrophic bacterium *Alkalilimnicola ehrlichii* sp. strain MLHE-1, *arxA* is required for anaerobic arsenite oxidation coupled to nitrate (12). The BSL-9 genome lacks the AioA-type arsenite oxidase. A BSL-9 *arxA* mutant strain shows that *arxA* is the sole arsenite oxidase for photoarsenotrophy (13).

*Ectothiorhodospiraceae* are common anoxygenic phototrophs with versatile abilities to metabolize inorganic and organic electron donors (14–16), which enables them to occupy distinct euphotic hypersaline alkaline environments. In addition to arsenite, BSL-9 can grow photoautotrophically with sulfide or thiosulfate. This is consistent with the presence of *sox* and *dsr* genes, which are involved in sulfur oxidation (17, 18). Moreover, BSL-9 can also grow as a photoheterotroph with various organic acids (e.g., acetate, malate, propionate, lactate, fumarate, succinate, and pyruvate). BSL-9 is sensitive to chloramphenicol, resistant to kanamycin, carbenicillin, gentamicin, and tetracycline, and grows optimally at 35°C at pH 8, 2% NaCl; these growth patterns are consistent with other *Ectothiorhodospira* species (14–16). Although BSL-9 is an anaerobe, the presence of cytochrome *c* oxidase genes (e.g. *cbb*_3_) found in BSL-9 may explain its tolerance to atmospheric oxygen. For example, cytochrome *c* oxidases (*cbb*_3_) are known for having high oxygen affinity, and cytochrome *c* peroxidases protect cells from reactive oxygen species. The BSL-9 genome also encodes photosynthetic complex genes, such as bacteriochlorophyll *a* synthase, the light-harvesting complex *pucAB*, and two copies of the carbon fixation-related gene *rbcL* (type III RuBisCO). Having the full genome sequence of BSL-9 opens numerous possibilities for studying the metabolic abilities, physiology, and the ecological environmental impact of photoarsenotrophy to the arsenic biogeochemical cycle.

The genome was done at the UC Davis Genome Sequencing Center using PacBio technology. The Hierarchical Genome Assembly Process (HGAP_v2) assembly pipeline (19) was used with ~300× sequence coverage. For annotation, the NCBI Public Genome Annotation Pipeline service was used. The resulting assembly was 3.5 Mb, with 63% G+C content.

### Accession number(s).

The genome sequence of *Ectothiorhodospira* sp. strain BSL-9 was deposited in the GenBank database under the accession no. CP011994, NCBI BioProject accession no. PRJNA232800, and BioSample accession no. SAMN03795182.

## References

[1] Oremland RS, Kulp TR, Blum JS, Hoeft SE, Baesman S, Miller LG, Stolz JF 2005 A microbial arsenic cycle in a salt-saturated, extreme environment. Science 308:1305–1308. doi:10.1126/science.1110832. 15919992

[2] Lloyd JR, Oremland RS 2006 Microbial transformations of arsenic in the environment: from soda lakes to aquifers. Elements 2:85–90. doi:10.2113/gselements.2.2.85.

[3] Hoeft SE, Blum JS, Stolz JF, Tabita FR, Witte B, King GM, Santini JM, Oremland RS 2007 *Alkalilimnicola ehrlichii* sp. nov., a novel, arsenite-oxidizing haloalkaliphilic gammaproteobacterium capable of chemoautotrophic or heterotrophic growth with nitrate or oxygen as the electron acceptor. Int J Syst Evol Microbiol 57:504–512. doi:10.1099/ijs.0.64576-0. 17329775

[4] Kulp TR, Hoeft SE, Asao M, Madigan MT, Hollibaugh JT, Fisher JC, Stolz JF, Culbertson CW, Miller LG, Oremland RS 2008 Arsenic(III) fuels anoxygenic photosynthesis in hot spring biofilms from Mono Lake, California. Science 321:967–970. doi:10.1126/science.1160799. 18703741

[5] Stolz JF, Basu P, Oremland RS 2010 Microbial arsenic metabolism: New twists on an old poison. Microbe 5:53–59. doi:10.1128/microbe.5.53.1.

[6] Edwardson CF, Planer-Friedrich B, Hollibaugh JT 2014 Transformation of monothioarsenate by haloalkaliphilic, anoxygenic photosynthetic purple sulfur bacteria. FEMS Microbiol Ecol 90:858–868. doi:10.1111/1574-6941.12440. 25318694

[7] Cloern JE, Cole BE, Oremland RS 1983 Seasonal changes in the chemistry and biology of a meromictic lake (Big Soda Lake, Nevada, USA). Hydrobiologia 105:195–206. doi:10.1007/BF00025188.

[8] Cloern JE, Cole BE, Oremland RS 1983 Autotrophic processes in meromictic Big Soda Lake, Nevada. Limnol Oceanogr 28:1049–1061. doi:10.4319/lo.1983.28.6.1049.

[9] Priscu JC, Axler RP, Carlton RG, Reuter JE, Arneson PA, Goldman CR 1982 Vertical profiles of primary productivity, biomass and physico-chemical properties in meromictic Big Soda Lake, Nevada, USA. Hydrobiologia 96:113–120. doi:10.1007/BF02185426.

[10] Zehr JP, Harvey RW, Oremland RS, Cloern JE, George LH, Lane JL 1987 Big Soda Lake (Nevada). 1. Pelagic bacterial heterotrophy and biomass. Limnol Oceanogr 32:781–793. doi:10.4319/lo.1987.32.4.0781.

[11] Zargar K, Conrad A, Bernick DL, Lowe TM, Stolc V, Hoeft S, Oremland RS, Stolz J, Saltikov CW 2012 ArxA, a new clade of arsenite oxidase within the DMSO reductase family of molybdenum oxidoreductases. Environ Microbiol 14:1635–1645. doi:10.1111/j.1462-2920.2012.02722.x. 22404962

[12] Zargar K, Hoeft S, Oremland R, Saltikov CW 2010 Identification of a novel arsenite oxidase gene, *arxA*, in the haloalkaliphilic, arsenite-oxidizing bacterium *Alkalilimnicola ehrlichii* strain MLHE-1. J Bacteriol 192:3755–3762. doi:10.1128/JB.00244-10. 20453090 PMC2897359

[13] Hernandez-Maldonado J, Sanchez-Sedillo B, Stoneburner B, Boren A, Miller L, McCann S, Rosen M, Oremland RS, Saltikov CW 2016 The genetic basis for anoxygenic photosynthetic arsenite oxidation. Environ Microbiol in press. doi:10.1111/1462-2920.13509. PMC5967609 27555453

[14] Trüper HG 1968 *Ectothiorhodospira mobilis* Pelsh, a photosynthetic sulfur bacterium depositing sulfur outside the cells. J Bacteriol 95:1910–1920. 5650091 10.1128/jb.95.5.1910-1920.1968 PMC252227

[15] Imhoff JF, Tindall BJ, Grant WD, Trper HG 1981 *Ectothiorhodospira vacuolata* sp. nov., a new phototrophic bacterium from soda lakes. Arch Microbiol 130:238–242. doi:10.1007/BF00459526.

[16] Gorlenko VM, Bryantseva IA, Rabold S, Tourova TP, Rubtsova D, Smirnova E, Thiel V, Imhoff JF 2009 *Ectothiorhodospira variabilis* sp. nov., an alkaliphilic and halophilic purple sulfur bacterium from soda lakes. Int J Syst Evol Microbiol 59:658–664. doi:10.1099/ijs.0.004648-0. 19329583

[17] Friedrich CG, Bardischewsky F, Rother D, Quentmeier A, Fischer J 2005 Prokaryotic sulfur oxidation. Curr Opin Microbiol 8:253–259. doi:10.1016/j.mib.2005.04.005. 15939347

[18] Frigaard N-U, Dahl C 2009 Sulfur metabolism in phototrophic sulfur bacteria. Adv Microb Physiol 54:103–200. doi:10.1016/S0065-2911(08)00002-7. 18929068

[19] Chin C-S, Alexander DH, Marks P, Klammer AA, Drake J, Heiner C, Clum A, Copeland A, Huddleston J, Eichler EE, Turner SW, Korlach J 2013 Nonhybrid, finished microbial genome assemblies from long-read SMRT sequencing data. Nat Methods 10:563–569. doi:10.1038/nmeth.2474. 23644548

